# Supplementary material for: Comparative Transcriptomics Provides Insight into the Neuroendocrine Regulation of Spawning in the Black-Lip Rock Oyster (Saccostrea echinata)
Source: Int J Mol Sci. 2025 Oct 15;26(20):10032. doi: 10.3390/ijms262010032 (PMC12564202; doi:10.3390/ijms262010032)
Supplement: Supplementary file 1 [file ijms-26-10032-s001.zip › Figure S1 &S2.pdf]

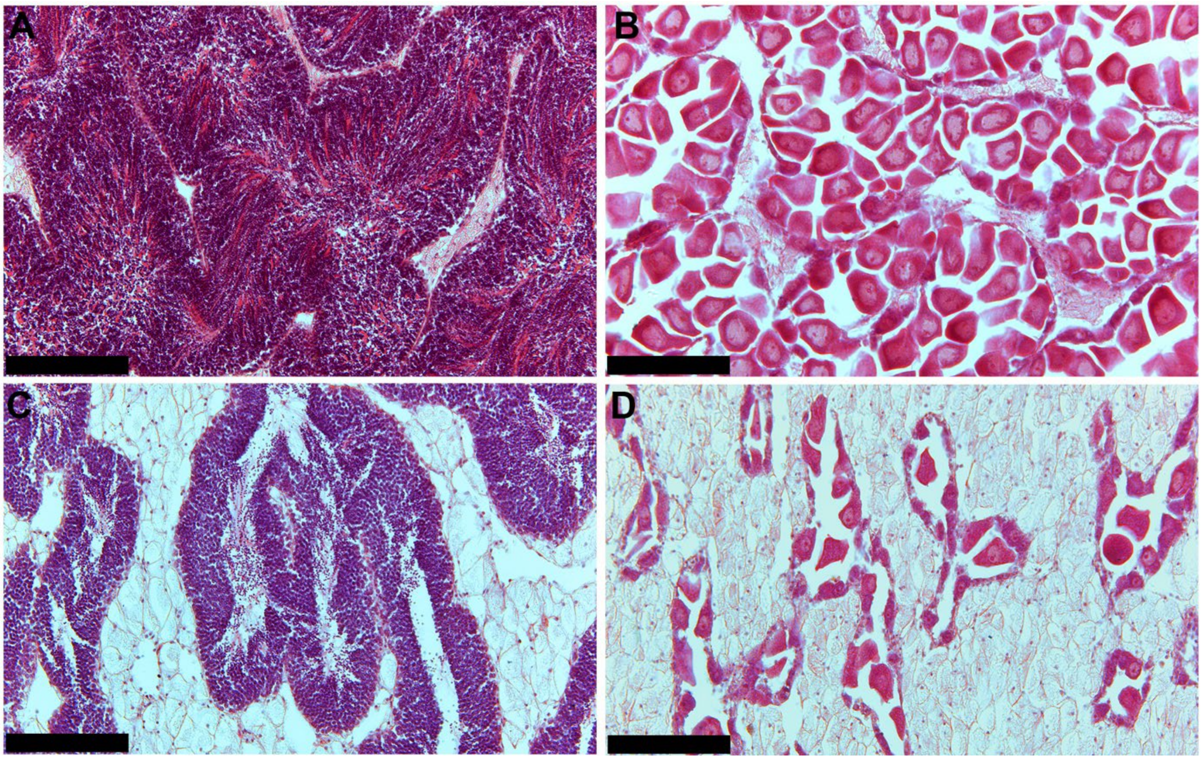

Figure S1. Histological sections of *S. echinata* male and female gonads. (**A)** Mature testis with gonadal follicles occupying a large area of the gonad, an absence of interstitial connective tissue and a lumen filled with spermatozoa. Spermatocytes are located near the follicular wall. **(B)** Mature ovary packed with fully matured ova and a thin layer of interstitial connective tissue. In the germinal epithelium, small primary oocytes are present. (**C)** Partially spawned testis with vacant spaces. (**D)** Partially spawned ovary with loosely packed ova and vacant spaces due to the absence of mature oocytes in the lumen of the follicle. Scale bars = 100 µm.

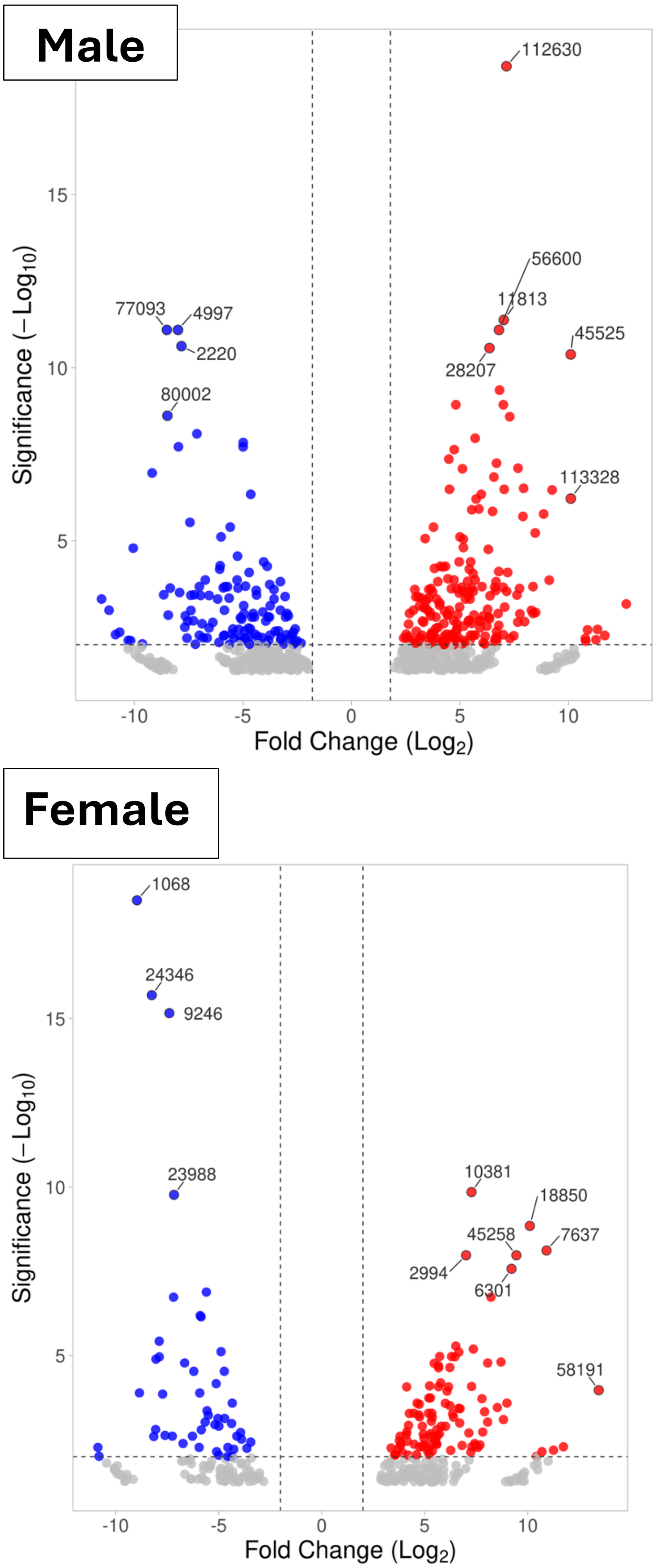

Figure S2. Volcano plots for upregulated DEGs in pre- and post-spawn male and female visceral ganglia.
