# Supplementary material for: Comparative Transcriptomics Provides Insight into the Neuroendocrine Regulation of Spawning in the Black-Lip Rock Oyster (Saccostrea echinata)
Source: Int J Mol Sci. 2025 Oct 15;26(20):10032. doi: 10.3390/ijms262010032 (PMC12564202; doi:10.3390/ijms262010032)
Supplement: Supplementary file 1 [file ijms-26-10032-s001.zip › Table S1.pdf]

Table S1. Summary statistics of *S. echinata* gonad and visceral ganglia (vg) RNA sequencing. Pre, ripe; Post, partial spent; F, female; M, male.

Items	Raw reads	Clean reads	Mapping reads	Mapping frequency	Q30
Pre gonad_F1	22,030,140	22,030,088	16,237,790	73.71	92.99
Pre gonad_F2	21,068,424	21,068,354	14,313,848	67.94	93.23
Pre gonad_F3	21,373,228	21,373,170	15,107,608	70.68	93.10
Pre gonad_M1	25,582,856	25,582,730	17,478,402	68.32	93.68
Pre gonad_M2	27,511,554	27,511,480	17,856,814	64.91	93.37
Pre gonad_M3	27,180,534	27,180,396	17,853,640	65.69	93.61
Pre vg_F1	21,014,196	21,014,138	13,128,580	62.47	93.41
Pre vg_F2	27,636,254	27,636,132	18,008,394	65.16	93.19
Pre vg_F3	22,420,106	22,420,034	14,566,734	64.97	92.92
Pre vg_M1	20,957,464	20,957,434	14,146,156	67.50	95.00
Pre vg_M2	20,327,546	20,327,488	13,362,952	65.74	93.41
Pre vg_M3	21,003,378	21,003,322	13,747,044	65.45	93.42
Post gonad_F1	27,470,118	27,469,972	17,854,988	65.00	94.24
Post gonad_F2	20,367,654	20,367,456	13,339,116	65.49	93.68
Post gonad_F3	21,635,286	21,635,064	13,133,568	60.71	93.49
Post gonad_M1	26,285,672	26,285,570	17,027,106	64.78	93.63
Post gonad_M2	27,2483,62	27,248,156	18,706,216	68.65	94.09
Post gonad_M3	26,517,962	26,517,776	17,876,958	67.41	93.92
Post vg_F1	21,384,622	21,384,568	13,708,158	64.10	93.42
Post vg_F2	25,722,238	25,722,138	15,442,528	60.04	91.77
Post vg_F3	25,900,866	25,900,744	16,619,912	64.17	93.41
Post vg_M1	25,366,086	25,366,022	16,366,164	64.52	93.56
Post vg_M2	28,916,550	28,916,410	18,383,962	63.58	93.40
Post vg_M3	21,065,622	21,065,558	14,029,494	66.60	93.35
