# Supplementary material for: Comparative Transcriptomics Provides Insight into the Neuroendocrine Regulation of Spawning in the Black-Lip Rock Oyster (Saccostrea echinata)
Source: Int J Mol Sci. 2025 Oct 15;26(20):10032. doi: 10.3390/ijms262010032 (PMC12564202; doi:10.3390/ijms262010032)
Supplement: Supplementary file 1 [file ijms-26-10032-s001.zip › Table_S2.pdf]

Table S2. Statistics of assembly and annotation for *S. echinata*.

Dataset name	Category	Number
Assembly	Number of transcripts	206,944
	Mean length of transcripts (bp)	581
	N50 length of transcripts (bp)	740
	Number of unigenes	191,197
	Mean length of transcripts (bp)	596
	N50 (bp) length of unigenes	781
BUSCO completeness	Complete BUSCO	58.3%
	Single-copy BUSCO	57.07%
	Duplicated BUSCO	1.23%
	Fragmented BUSCO	13.14%
	Missing BUSCO	28.56%
	Total BUSCO searched	5200
